# Distinguishing repeated polymerase chain reaction positivity from re‐infections in COVID‐19

**DOI:** 10.1111/irv.12883

**Published:** 2021-07-23

**Authors:** Sara Sadr, Melika Arab Bafrani, Alireza Abdollahi, Seyed Ahmad SeyedAlinaghi, Esmaeil Mohammadnejad, Roghieh Hossienzade, Fereshteh ShahmariGolestan, Zahra Ahmadinejad, Mohamadreza Salehi, Mohammad Javaherian, Elahe Kimyaee, Fatemeh Jafari, Fereshteh Ghiasvand

**Affiliations:** ^1^ School of Medicine Mazandaran University of Medical Sciences Sari Iran; ^2^ Students^'^ Scientific Research Center (SSRC), School of Medicine Tehran University of Medical Sciences Tehran Iran; ^3^ Department of Pathology, School of Medicine, Imam Khomeini Hospital Complex Tehran University of Medical Sciences Tehran Iran; ^4^ Iranian Research Center for HIV/AIDS, Iranian Institute for Reduction of High Risk Behaviors Tehran University of Medical Sciences Tehran Iran; ^5^ Nursing Education, Department of Medical‐Surgical Nursing and Basic Science, School of Nursing and Midwifery Tehran University of Medical Sciences Tehran Iran; ^6^ Imam Khomeini Hospital Complex Tehran University of Medical Sciences Tehran Iran; ^7^ Department of Infectious Diseases Tehran University of Medical Sciences Tehran Iran; ^8^ Liver Transplantation Research Center, Department of Infectious Diseases, Imam Khomeini Hospital Complex Tehran University of Medical Sciences Tehran Iran; ^9^ Department of Infectious Diseases, Imam Khomeini Hospital Complex Tehran University of Medical Sciences Tehran Iran; ^10^ Department of Physiotherapy, School of Rehabilitation, Liver Transplantation Research Center, Imam Khomeini Hospital Complex Tehran University of Medical Sciences Tehran Iran; ^11^ Central Laboratory, Imam Khomeini Hospital Complex Tehran University of Medical Sciences Tehran Iran; ^12^ School of Medicine Iran University of Medical Sciences Tehran Iran

**Keywords:** COVID‐19, polymerase chain reaction, reinfection, seroconversion, virus shedding

## Abstract

**Background:**

Possibility of reinfection with SARS‐CoV‐2 changes our view on herd immunity and vaccination and can impact worldwide quarantine policies. We performed real‐time polymerase chain reaction (RT‐PCR) follow‐up studies on recovered patients to assess possible development of reinfections and re‐positivity.

**Methods:**

During a 6‐month period, 202 PCR‐confirmed recovering COVID‐19 patients entered this study. Follow‐up RT‐PCR tests and symptom assessment were performed 1 month after the initial positive results. Patients who tested negative were tested again 1 and 3 months later. The serum IgG and IgM levels were measured in the last follow‐up session.

**Results:**

In the first two follow‐up sessions, 82 patients continued their participation, of which four patients tested positive. In the second follow‐up 44 patients participated, three of whom tested positive. None of the patients who tested positive in the first and second follow‐up session were symptomatic. In the last session, 32 patients were tested and four patients were positive, three of them were mildly symptomatic and all of them were positive for IgG.

**Conclusions:**

A positive RT‐PCR in a recovering patient may represent reinfection. While we did not have the resources to prove reinfection by genetic sequencing of the infective viruses, we believe presence of mild symptoms in the three patients who tested positive over 100 days after becoming asymptomatic, can be diagnosed as reinfection. The immune response developed during the first episode of infection (e.g., IgG or T‐cell mediated responses that were not measured in our study) may have abated the symptoms of the reinfection, without providing complete protection.

## 1 INTRODUCTION

Since November 2019, the Severe Acute Respiratory Syndrome Coronavirus 2 (SARS‐CoV‐2) has infected more than 134 million people worldwide and has caused more than 2 million deaths.^1^ No curative drug or specific treatment is known to have considerable efficacy against this infection; but, currently, over 200 candidate vaccines have been acknowledged by the WHO.^2^, ^3^, ^4^

Our experience with other respiratory infections, such as those caused by influenza and seasonal respiratory infections caused by coronavirus lead us to believe that the natural immunity after an episode of infection with this virus may not cause long‐time immunity.^5^ Some studies have shown a substantial wane in antibody levels within a few months after remission^6^, ^7^; and the studies that report re‐infection further question the efficacy and longevity of the acquired immunity after infection with this virus. Several cases of suspected and proven instances of re‐infection with SARS‐CoV‐2 have been reported in immunocompetent patients in different age groups, in patients with different levels of antibody response.^8^, ^9^, ^10^

The duration of sustained antibody response after an episode of infection is generally a representative of the immune protection that can be achieved by vaccination against the same virus.^11^ Waning of the IgG antibodies that are naturally produced in a patient after infection with SARS‐CoV‐2, undermines the supposed efficacy of vaccination; especially since cases of symptomatic re‐infection with replication‐competent virus have been reported within the first 6 months after the initial infection.^12^, ^13^

To this date, thousands of distinct variants of SARS‐CoV‐2 have been identified with over 400 variants in the spike protein, which is presumably the binding site of neutralizing antibodies.^14^ Besides, in many cases of re‐infection, genetic sequencing has revealed a different clade of the virus to be the causative pathogen.^15^, ^16^, ^17^, ^18^ We can assume—as is the case with infections caused by rhinoviruses and influenza—that the protective activity of antibodies is limited to each specific subtype of the virus, this can possibly explain why re‐infection can occur in presence of detectable levels of IgG.^19^, ^20^

In this study we investigated the rate of symptomatic and asymptomatic re‐positivity with SARS‐CoV‐2 in recovering patients for up to 4 months after the initial diagnosis of Coronavirus Disease 2019 (COVID‐19); and find possible risk factors that are associated with re‐infection.

## 2 METHODS

### 2.1 Study design and participants

This prospective study was performed for a duration of 6 months from May to September of 2020 on Coronavirus Disease 2019 (COVID‐19) patients who were admitted to the Imam Khomeini Referral Hospital, Tehran, Iran.

### 2.2 Eligibility criteria

Patients who had been diagnosed with COVID‐19 (approved by a real‐time polymerase chain reaction [RT‐PCR] test of nasopharyngeal specimens) and had been admitted based on the national criteria for hospitalization (a sustained peripheral oxygen saturation of under 93% and/or a respiratory rate of over 30/min or sustained nausea and vomiting and severe weakness even with normal oxygen saturation), were brought into the study upon discharge. The current national discharge criteria dictate that prior to discharge patients must have at least two consecutive afebrile days with a blood oxygen saturation of over 90%, oral intake without nausea, and improvement in weakness. The exclusion criteria were a negative RT‐PCR result or lack of documentation of a positive result at the initial hospitalization.

### 2.3 Study initiation and follow‐up sessions

All information regarding patients' admission and epidemiological data including age, sex, any past medical history, and recent use of immunocompromising medications were documented.

**FIGURE 1:**
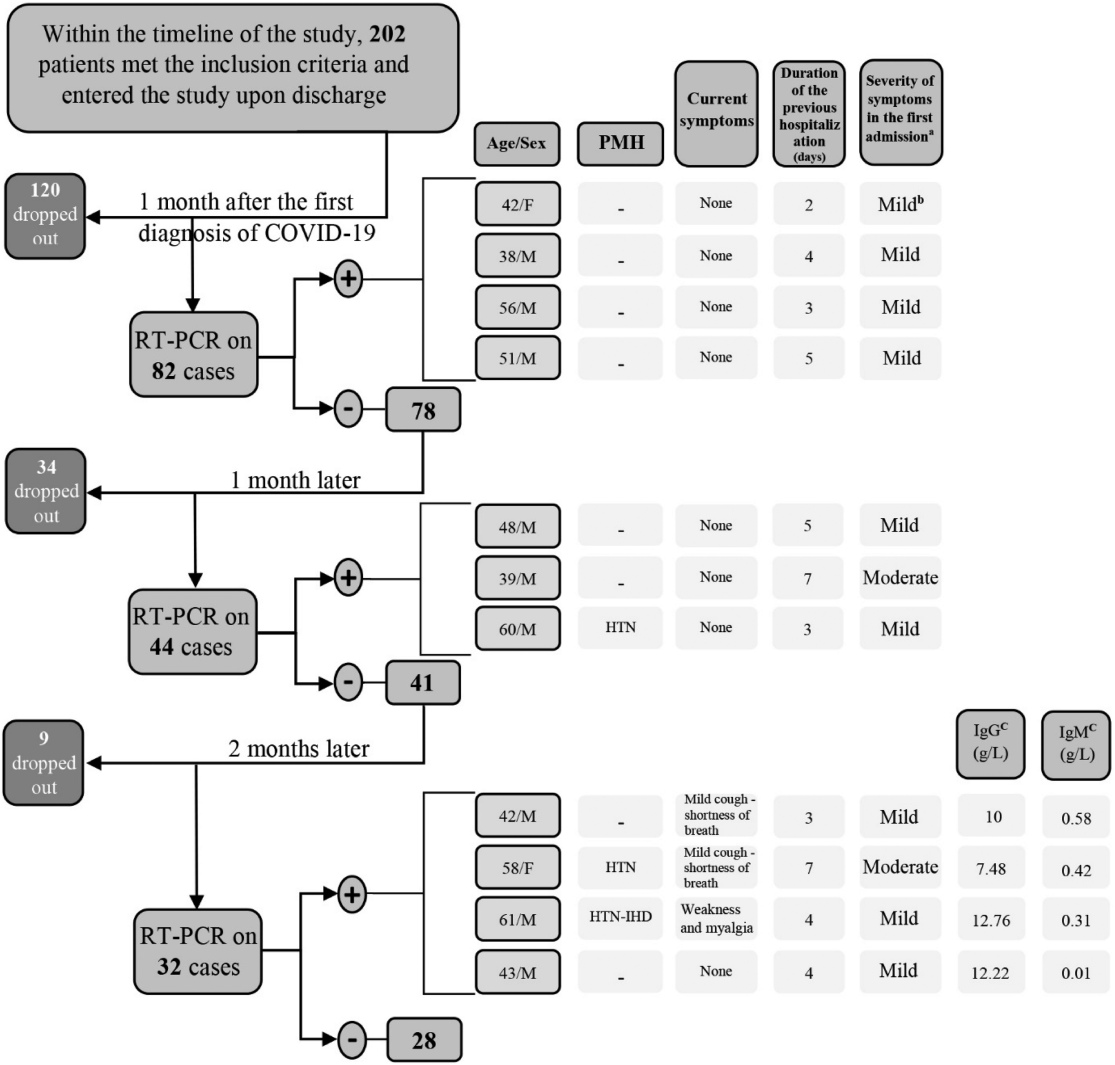
Detailed information on real‐time polymerase chain reaction (RT‐PCR)‐positive patients along the study time‐line. (A) Patients with a sustained peripheral oxygen saturation (O2Sat) of under 90% were considered severe cases, patients with an O2Sat of between 90% and 93% were considered moderate, and patients with higher levels of O2Sat were mild. (B) Patients with a high O2Sat were admitted if they experienced other considerably impairing symptoms; which in case of all these patients were severe nausea and vomiting or excessive weakness. (C) Levels lower than 1.1 g/L are considered negative. (D) Past medical history. (E) Hypertension. (F) Ischemic heart disease

The first follow‐up RT‐PCR study was performed 1 month after the initial positive RT‐PCR test; at which point all patients had been asymptomatic for at least 14 days. All patients who tested negative were re‐tested 1 and 3 months after the date of the first follow‐up RT‐PCR test (Figure 1). A complete assessment of signs and symptoms related to COVID‐19 along with serology testing for anti‐SARS‐CoV‐2 IgG and IgM levels were also performed during the third follow‐up visit.

### 2.4 Real‐time polymerase chain reaction

A nasopharyngeal swab sample was obtained by a trained technician and RNA was extracted with a Real Genomics viral nucleic acid extraction kit (Cat.No.YVN50/YVN100). The Novel Coronavirus (2019‐nCOV) Nucleic Acid Diagnostic Kit (PCR‐Fluorescence Probing), Sansure Biotech (S3102E), made in Changsha, China was used for qualitative detection of the ORF1ab and N genes of 2019‐nCOV with a cycle threshold of less than 35 (Ct < 35) for positive control and Ct > 40 for negative control at channel FAM, ROX and CY5 (internal control) according to kit instructions.

### 2.5 Antibodies

A 5 cc whole blood sample was drawn (without anticoagulants), and serum was derived from the specimen using centrifugation (3000xg for 10 min). The Enzyme‐linked Immunosorbent Assay (ELISA) method (Pishtaz Teb SARS‐CoV‐2 IgM and IgG Iran) was used to test the serum antibody levels. The test was performed according to the manufacturer's brochure. Results greater than 1.1 were considered positive and those less than 0.9 as negative. Results within the mentioned range were reported as borderline and the test was redone on a second, fresh serum sample to confirm the initial results.

The manufacture reported diagnostic specifications of the test kits:

Specificity: 97.30% and Sensitivity: 79.40% for SARS‐COV‐2‐IGM kit.

Specificity: 98.30%% and sensitivity: 91.10% for SARS‐COV‐2‐IGG kit.

### 2.6 Measurements and statistical analysis

Data were analyzed using SPSS software V.22.0. Quantitative variables are reported by mean and standard deviation (SD) and qualitative variables are reported using frequency and percentage. Chi‐square and Fisher's exact tests were used to assess the statistical relationships between categorical variables. The level of significance was set as *P* value < .05 for all analyses.

### 2.7 Ethical considerations

Informed consent was obtained from participants and they were advised that they can leave the study at any time point and this will not hinder their current treatment or future visits to the hospital and the quality of care they would receive. This study was conducted in accordance with the 1964 Helsinki Declaration and approved by the Tehran University of Medical Sciences ethics board committee (Ethics code: IR.TUMS.VCR.REC.1399.076).

## 3 RESULTS

### 3.1 One month after the first positive real‐time polymerase chain reaction (1st F/U)

Overall, 202 discharged patients were contacted for follow‐up laboratory studies and 82 patients, including 27 women (33%) and 55 men (64%), decided to participate in this study.

Patients had a median age of 47 ranging between 29 and 84 years old. Table 1 shows the demographic description of participants. The first RT‐PCR results of four patients (4.87%) were positive; who were asymptomatic and not different from those who tested negative in terms of the duration of the initial admission, the severity of the first episode of the disease, presence and type of underlying diseases, and recent history of using immunosuppressive drugs (*P* value: .63, .57, .59, and .61).

**TABLE 1:** Demographic data of participants

		1st F/U	2nd F/U	3rd F/U
**Number of patients**		82	44	32
**Female**		25	16	12
**Male**		57	28	20
**Age**		52 ± 14	49 ± 16	47 ± 16
**Underlying disease** ^a^ **:**				
	**DM**	12	9	5
	**HTN**	27	21	13
	**Cardiovascular dis.**	5	2	0
	**Chronic pulmonary dis.**	3	1	1
	**Malignancies**	2	0	0
	**AIDS**	1	1	0
	**Smoker**	11	8	7
	**Recent use of immunosuppressive agents**	1	0	0

^a^ No patients in our study had a history of ESRD (End‐Stage Renal Disease), chronic liver disease, neurological disorders, morbid obesity, transplantation or any other immune‐compromising condition other than AIDS. DM (Diabetes mellitus), HTN (Hypertension), AIDS (Acquired immunodeficiency syndrome).

### 3.2 Two months after the first positive real‐time polymerase chain reaction (2nd F/U)

One month after the first negative follow‐up result, among the 78 patients who had tested negative, 44 patients (53.65%) continued their participation in the study. Additional data regarding patients who tested positive are presented in Figure 1. The patients who tested positive were not different in terms of the duration of the initial admission, severity of the disease, and the need for ICU admission (*P* value: .69, .62, and .65).

### 3.3 Four months after the first positive real‐time polymerase chain reaction (3rd F/U)

**A**mong the 41 patients who had tested negative, 32 patients (39.02%) continued their participation in the study. Four patients had a positive RT‐PCR result (12.5%). None of the patients who tested positive were symptomatic in previous studies; but now three of them were mildly symptomatic; also, two patients had underlying medical conditions (Figure 1). None of the patients who tested negative were symptomatic. The underlying medical conditions, history of using immunosuppressive drugs, initial disease severity, admission duration and history of ICU admission in the first episode of infection were not significantly different between patients who had a positive RT‐PCR result and those who did not (*P* value: .67, .59, .64, .64, and .71).

The IgM levels were <1.1 g/L in 30 (94%) patients (negative); and positive in two patients. The patients who tested positive were asymptomatic and did not have a positive RT‐PCR test in any of the previous follow‐up studies. The IgG levels were >1.1 g/L in 30 (94%) patients (positive); and negative in two patients. The mean IgG level was 7.94 ± 3.67 g/L and ranged between 1.48 and 14.5 g/L. The two patients, who had a negative IgG test result, did not test positive in any of the follow‐up RT‐PCR tests.

All patients recovered with no further complications and none of the patients who re‐tested positive required readmission or further medical treatment.

## 4 DISCUSSION

### 4.1 Interpretations of a positive real‐time polymerase chain reaction re‐test

Considering the high infection rate, few cases of reinfection with SARS‐CoV‐2 have been reported. Often, for the diagnosis COVID‐19 reinfection a positive RT‐PCR test with or without accompanying symptoms has been used. Although, in these cases different speculations for interpretation of a re‐positivity can be made; each reinfection of which will have specific reverberations.^21^

In some studies, a positive RT‐PCR in a recovering patient who tested negative upon resolution of their symptoms has been considered a strong indicator of.^10^ Although, false results are possible. A false positive result can occur in a recovering patient; also a false negative result shortly after subsidence of symptoms, followed by a correctly positive test misleads physicians toward a re‐infection diagnosis.^22^, ^23^, ^24^ To avoid this problem—as per WHO recommendations—in many regions the treatment protocol for COVID‐19 requires two consecutive negative RT‐PCR results prior to discharge.^25^, ^26^

Also, RT‐PCR cannot differentiate replication‐competent viruses from viral fragments that are expelled from a recovering patient.^27^, ^28^ Viral shedding from the respiratory tract during recovery has been reported to last for as long as 12 weeks after infection^29^; thus, a significant time‐gap between the first episode of infection and a positive RT‐PCR can clarify that the patient has passed the viral‐shedding stage.

In this study RT‐PCR testing was not performed upon discharge to confirm viral clearance. The first follow‐up RT‐PCR study was performed 30 days after the first positive RT‐PCR result in each patient, which was after at least 14 days of being asymptomatic. Considering the short interval, lack of proof of prior viral clearance, and the fact that the patients who tested positive were asymptomatic, we believe that the positive results show continued viral shedding rather than re‐infection. Other studies have reported that up to 14% of recovering asymptomatic patients who tested negative upon discharge, re‐test positive.^30^ Symptomatic cases of re‐positivity have also been reported. A 24‐year‐old health‐worker became symptomatic and tested positive for SARS‐CoV‐2 within 52 days of an initial diagnosis of COVID‐19. Similar to our study, no confirmatory RT‐PCR testing was performed at discharge. Serum antibodies were not detected at the beginning of the second symptomatic period, which can represent an incomplete immune response that left the infection temporarily dormant, only to be re‐activated again; and although the patient was symptomatic and had continued contact with infected patients, re‐activation was considered more probable than re‐infection.^31^ Similar studies have reported a return of mild or even severe symptoms; but within a short time frame and without genetic analysis of the infective pathogens, they were reported to be cases of reactivation rather than re‐infection.^32^, ^33^, ^34^, ^35^

Three patients (6.81%) tested positive 60 days after the initial infection (1 month after the first negative RT‐PCR test). These patients again were asymptomatic and the interval between the initial infection and this positive test is not significant enough to rule out viral shedding. Although, in case of viral shedding, we would have expected to achieve a positive result in the previous test as well as this one; but this dissonance can be explained in absence of re‐infection. Given the limited accuracy of RT‐PCR, in case of a false negative in the first follow‐up test or a false positive in the second test, continued shedding or complete recovery (respectively) can be misdiagnosed as re‐infection.^36^, ^37^ On the other hand, studies have shown that COVID‐19 patients have a lower concentration of ACE2 monocyte expression—the endogenous entry receptor of SARS‐CoV‐2—and researchers hypothesize that the virus can remain dormant in peripheral blood mononuclear cells and cause a relapse after the respiratory system has been cleared of the virus and patient has tested negative.^38^, ^39^, ^40^

Positive RT‐PCR tests in absence of significant symptoms in recovering patients have also been reported in many studies.^18^, ^41^ In some cases, symptoms were present, although less severe than the first episode.^42^ Contrasting our results, the majority of other reports have described more severe symptoms in patients who re‐tested positive^15^, ^16^, ^17^, ^43^ and researchers have hypothesized that a selection bias toward testing and confirming re‐infection in symptomatic patients^44^ and/or a primed/heightened immune response upon the second episode of infection can be the reason why most cases of re‐infection pertain to patients with intense symptoms upon second exposure and infection.^15^, ^43^

An episode of re‐infection can be proved by a positive viral culture^21^, ^45^ and/or genetic sequencing of the infective virus in both episodes to show infection with another sub‐class of the virus. Tillett et al., reported a case of re‐infection in a 25‐year‐old male, who recovered from a RT‐PCR‐confirmed episode of COVID‐19, only to become symptomatic again after a 30‐day symptom‐free period; which genetic sequencing proved to be caused by a different variant of the virus.^15^ Similar cases of re‐infection within 6 months of an original episode of COVID‐19 have been reported.^16^, ^17^, ^18^

Based on CDC recommendations in absence of genetic proof of infection with a different clade of the virus, a positive RT‐PCR test that has been obtained after the first 90 days of the onset of the initial infection can be considered indicative of re‐infection. Although, a positive RT‐PCR test after two consecutive negative results, especially if accompanied by typical symptoms, can be defined as re‐infection even within the first 90 days of the first episode of infection.^21^

Based on the recommended CDC definitions,^29^ re‐infection is a probable diagnosis for the three symptomatic patients who tested positive in the last RT‐PCR test; but it is less likely in case of the one asymptomatic patient. The third RT‐PCR screening was performed 120 days after the diagnosis of COVID‐19 in 32 patients. Although, the symptoms could have been caused by re‐activation of dormant infection and release of viruses from body reservoirs, they could also be caused by re‐infection. A similar process involving latent infection of cells followed by transcription of viral genome has also been suggested, which would result in reactivation of the virus from a latent to a lytic stage after a symptom‐free period, causing a resurgence of COVID‐19 symptoms^40^; but the long interval between the two positive RT‐PCR results makes re‐activation an unlikely diagnosis.^21^ We did not find any risk factors that could help distinguish patients who are more susceptible to re‐infection from those who are not.

### 4.2 Humoral response

Both insufficient and overactive immune responses have been reported in COVID‐19 patients.^46^ The dynamics of the antibody response in COVID‐19 patients is not completely understood; and different rates of seroconversion have been reported. Zhao et al.^47^ and Liu et al.^48^ reported seroconversion in all infected patients respectively by 39 and 14 days after the onset of infection. Liu et al. also reported that by the 60th day IgM antibodies were undetectable in about one‐third of the patients and the IgG titers had decreased substantially.^48^ Another study showed recently discharged patients have an even high levels of antibodies start to decrease within 2 to 3 months after the infection.^13^ In another study the seroconversion rate for IgG, IgM and IgA was ~90% and most patients seroreverted within 75 days; with IgG levels remaining detectable over 90 days after the symptom onset in more than 99% of patients.^49^ Multiple studies have also concluded that the humoral immunity against this virus could be short‐lived.^50^ Contrasting these studies, our results showed that 94% of patients were positive for neutralizing antibodies (IgG) 120 days after the onset of symptoms; which is in line with the results of an Icelandic population study that reported a 91% seropositivity 4 months after the initial diagnosis of COVID‐19.^6^ To evaluate these results, we should take into account the natural process of the humoral response. In case of many other viral infections—where seroconversion is sustained as seromaintenance and immunity—we see a temporary decrease of antibody levels during the first few months of infection/inoculation,^51^ and since the emergence of COVID‐19 is recent, we could expect a rebound increase in antibody levels later on.^50^

In our study, the four patients who had a positive result in RT‐PCR screening 120 days after the initial diagnosis of COVID‐19, were also positive for antibodies; and although they theoretically may have prevented a severe episode of re‐infection and caused a lack of any symptoms in one RT‐PCR‐positive patient, we cannot know for sure if those levels are high enough to be completely protective.^21^ In a similar study Zhang et.al reported re‐infection in six recovered patients that was caused by viruses from lineages different from the first infection. All these patients had varied levels of antibodies and they concluded that presence and even maintenance of the humoral response cannot rule out the possibility of re‐infection.^52^ We believe that the two patients who did not have sufficient levels of IgG (<1.1 g/L), have been protected from an episode of re‐infection by a strong cellular immune response, even within an epidemic situation.

In cases of re‐infection with a different clade of the virus, even protective levels of IgG may not be effective.^21^ We hypothesize that high levels of neutralizing antibodies do not make the diagnosis of re‐infection unlikely, unless there is genetic proof that the positive RT‐PCR results are related to the same strain of the virus from the first episode; in which case re‐activation/relapse would be a more likely diagnosis.

### 4.3 Limitations

Some of the limitations of our study were that a considerable number of participants dropped out during the study, this study was only performed in one medical center, and that we could not perform genetic sequencing to prove infection with a different clade of the virus, or cultures to prove presence of replication‐competent virus. The diagnosis of re‐infection in our study is based on RT‐PCR results, typical symptoms and the long interval between the two positive results. And, since we did not measure the viral load in patients who tested positive, the diagnosis of re‐infection in our study is as certain as is the specificity of the RT‐PCR test. We were also unable to perform required serology testing from the beginning of the study.

## 5 CONCLUSION

Based on current diagnostic guidelines, re‐infection associated with mild symptoms was detected in three out of 82 patients; barring possible false positive results. We cannot confirm re‐infection without positive viral cultures. We also believe that the quantity of antibodies that are produced against this virus can be sustained longer than the initial studies suggest, although the protective abilities of these antibodies against infection with the same or a different subtype of the virus needs to be studied further. The emergence of new vaccines against this virus is a considerable achievement with limited guaranteed outcomes, because the intricacies of the long‐term immune response against this virus is not fully known, and further studies on cases of supposed re‐infection are needed to clarify the probability and underlying risk factors of re‐infection.

## AUTHOR CONTRIBUTIONS

**Sara Sadr:** Writing‐original draft; writing‐review and editing. **Melika Arab Bafrani:** Investigation. **Alireza Abdollahi:** Conceptualization; investigation. **SeyedAhmad SeyedAlinaghi:** Formal analysis. **Esmaeil Mohammadnejad:** Data curation. **Roghieh Hossienzade:** Investigation. **Fereshteh Shahmari Golestan:** Data curation. **Zahra Ahmadinejad:** Investigation. **Mohammadreza Salehi:** Supervision. **Mohammad Javaherian:** Investigation. **Elahe kimyaee:** Methodology. **Fatemeh Jafari:** Data curation. **Fereshteh Ghiasvand:** Project administration.

## CONFLICT OF INTEREST

**N**one.

### PEER REVIEW

The peer review history for this article is available at https://publons.com/publon/10.1111/irv.12883.

## Data Availability

The data that support the findings of this study are available on request from the corresponding author. The data are not publicly available due to privacy and ethical restrictions.

## References

[1] World Health Organization , WHO Coronavirus Disease (COVID‐19) dashboard. 2021 February 4th, 2021; Available from: https://covid19.who.int/

[2] Shang W , Yang Y , Rao Y , Rao X . The outbreak of SARS‐CoV‐2 pneumonia calls for viral vaccines. Npj Vaccines. 2020;5(1):1‐3. 32194995 10.1038/s41541-020-0170-0 PMC7060195

[3] Ayotte, K. , J. Gerberding , and J.S. Morrison , Ending the cycle of crisis and complacency in US global health security: a report of the CSIS commission on strengthening America's health security. 2020: Center for Strategic & International Studies.

[4] WHO , Draft landscape of COVID‐19 candidate vaccines. World Health Organisation, 2020.

[5] Cohen JI , Burbelo PD . Reinfection with SARS‐CoV‐2: implications for VaccinesClinical Infectious Diseases. 2020. 10.1093/cid/ciaa1866 PMC7799323 33338197

[6] Gudbjartsson DF , Norddahl GL , Melsted P , et al. Humoral immune response to SARS‐CoV‐2 in Iceland. New Eng J Med. 2020;383(18):1724‐1734. 32871063 10.1056/NEJMoa2026116 PMC7494247

[7] Crawford KH , Dingens AS , Eguia R , et al. Dynamics of Neutralizing Antibody Titers in the Months After SARS‐CoV‐2 InfectionMedRxiv; 2020. 10.1093/infdis/jiaa618 PMC7543487 33535236

[8] Dickson M , Mathews R , Menon GG . Analysis of COVID‐19 Reinfection Rates and Its Underlying Causes: A Systematic Review; 2020.

[9] Krishna E , Pathak VK , Prasad R , Jose H , Kumar MM . COVID‐19 reinfection: linked possibilities and future outlook. J Fam Med Prim Care. 2020;9(11):5445. 10.4103/jfmpc.jfmpc_1672_20 PMC7842419 33532377

[10] Osman A , Al Daajani M , Alsahafi A . Re‐positive coronavirus disease 2019 PCR test: could it be a reinfection? New Microbes New Infect. 2020. 10.1016/j.nmni.2020.100748 PMC7439804 32843984

[11] Plotkin SA . Correlates of protection induced by vaccination. Clin Vaccine Immunol. 2010;17(7):1055‐1065. 20463105 10.1128/CVI.00131-10 PMC2897268

[12] Seow J , Graham C , Merrick B , et al. Longitudinal Evaluation and Decline of Antibody Responses in SARS‐CoV‐2 InfectionMedRxiv; 2020; 5(12): 1598–1607. 10.1038/s41564-020-00813-8 PMC7610833 33106674

[13] Long Q‐X , Tang XJ , Shi QL , et al. Clinical and immunological assessment of asymptomatic SARS‐CoV‐2 infections. Nat Med. 2020;26(8):1200‐1204. 32555424 10.1038/s41591-020-0965-6

[14] Koyama T , Platt D , Parida L . Variant analysis of SARS‐CoV‐2 genomes. Bull World Health Organ. 2020;98(7):495. 32742035 10.2471/BLT.20.253591 PMC7375210

[15] Tillett RL , Sevinsky JR , Hartley PD , et al. Genomic evidence for reinfection with SARS‐CoV‐2: a case study. Lancet Infect Dis. 2021;21(1):52‐58. 33058797 10.1016/S1473-3099(20)30764-7 PMC7550103

[16] Prado‐Vivar B , Becerra‐Wong M , Guadalupe JJ , et al. COVID‐19 re‐infection by a phylogenetically distinct SARS‐CoV‐2 variant, first confirmed event in South America. First confirmed event in South America. (September 3, 2020), 2020.

[17] Van Elslande J , Vermeersch P , Vandervoort K , et al. Symptomatic SARS‐CoV‐2 reinfection by a phylogenetically distinct strain. Clin Infect Dis. 2020;10. 10.1093/cid/ciaa1330 PMC7499557 32887979

[18] Scaria V . Asymptomatic Reinfection in Two Healthcare Workers From India With Genetically Distinct SARS‐CoV‐2; 2020.

[19] Mo H , Zeng G , Ren X , et al. Longitudinal profile of antibodies against SARS‐coronavirus in SARS patients and their clinical significance. Respirology. 2006;11(1):49‐53. 16423201 10.1111/j.1440-1843.2006.00783.x PMC7192223

[20] Payne DC , Iblan I , Rha B , et al. Persistence of antibodies against Middle East respiratory syndrome coronavirus. Emerg Infect Dis. 2016;22(10):1824‐1826. 27332149 10.3201/eid2210.160706 PMC5038413

[21] Yahav D , Yelin D , Eckerle I , et al. Definitions for coronavirus disease 2019 reinfection, relapse and PCR re‐positivity. Clin Microbiol Infect. 2020. 10.1016/j.cmi.2020.11.028 PMC7718119 33285276

[22] Luo A . Positive SARS‐Cov‐2 test in a woman with COVID‐19 at 22 days after hospital discharge: a case report. J Tradit Chin Med Sci. 2020;7(4):413‐417.

[23] SeyedAlinaghi S , Oliaei S , Kianzad S , et al. Reinfection risk of novel coronavirus (CoVID‐19): a systematic review of current evidence. World J Virol. 2020;9(5):79‐90. 33363000 10.5501/wjv.v9.i5.79 PMC7747024

[24] Arevalo‐Rodriguez I , Buitrago‐Garcia D , Simancas‐Racines D , et al. False‐negative results of initial RT‐PCR assays for COVID‐19: a systematic review. PloS One. 2020;15(12):e0242958. 33301459 10.1371/journal.pone.0242958 PMC7728293

[25] World Health Organization . Criteria for releasing COVID‐19 patients from isolation: scientific brief, 17 June 2020. 2020. World Health Organization

[26] Tao W , Wang X , Zhang G , et al. Re‐detectable positive SARS‐CoV‐2 RNA tests in patients who recovered from COVID‐19 with intestinal infection. Protein Cell. 2020;1‐6. 32978728 10.1007/s13238-020-00778-8 PMC7518948

[27] Kang H , Wang Y , Tong Z , Liu X . Retest positive for SARS‐CoV‐2 RNA of “recovered” patients with COVID‐19: persistence, sampling issues, or re‐infection? J Med Virol. 2020;92(11):2263‐2265. 32492212 10.1002/jmv.26114 PMC7300489

[28] Chen Y , Bai W , Liu B , et al. Re‐evaluation of retested nucleic acid‐positive cases in recovered COVID‐19 patients: report from a designated transfer hospital in Chongqing, China. J Infect Public Health. 2020;13(7):932‐934. 32540264 10.1016/j.jiph.2020.06.008 PMC7275981

[29] Centers for Disease Control and Prevention , Duration of isolation and precautions for adults with COVID‐19. 2020. 2020.

[30] Lu J , Peng J , Xiong Q , et al. Clinical, immunological and virological characterization of COVID‐19 patients that test re‐positive for SARS‐CoV‐2 by RT‐PCR. EBioMedicine. 2020;59:102960. 32853988 10.1016/j.ebiom.2020.102960 PMC7444471

[31] Bonifácio LP , Pereira APS , Araújo DCA , et al. Are SARS‐CoV‐2 reinfection and Covid‐19 recurrence possible? a case report from Brazil. Rev Soc Bras Med Trop. 2020;53. 10.1590/0037-8682-0619-2020 PMC7508196 32965458

[32] Ye G , Pan Z , Pan Y , et al. Clinical characteristics of severe acute respiratory syndrome coronavirus 2 reactivation. J Infect. 2020;80(5):e14‐e17. 10.1016/j.jinf.2020.03.001 PMC7102560 32171867

[33] Ravioli S , Ochsner H , Lindner G . Reactivation of COVID‐19 pneumonia: a report of two cases. J Infect. 2020. 10.1016/j.jinf.2020.05.008 PMC7204684 32389787

[34] Kang YJ , Joo H . South Korea's COVID‐19 infection status: from the perspective of reconfirmation after complete recovery. J Pure Appl Microbiol. 2020;14.

[35] Loconsole D , Passerini F , Palmieri VO , et al. Recurrence of COVID‐19 after recovery: a case report from Italy. Infection. 2020;1‐3. 10.1007/s15010-020-01444-1 PMC7228864 32415334

[36] Anwar H , Khan QU . Pathology and Therapeutics of COVID‐19: a review. Int J Med Stud. 2020;8(2):113‐120.

[37] Xie C , Lu J , Wu D , et al. False negative rate of COVID‐19 is eliminated by using nasal swab test. Travel Med Infect Dis. 2020. 10.1016/j.tmaid.2020.101668 PMC7151360 32283215

[38] Zhang D , Guo R , Lei L , et al. COVID‐19 infection induces readily detectable morphologic and inflammation‐related phenotypic changes in peripheral blood monocytes. J Leukoc Biol. 2020. 10.1002/JLB.4HI0720-470R PMC7675546 33040384

[39] Elberry MH , Ahmed H . Occult SARS‐CoV‐2 infection; a possible hypothesis for viral relapse. Med Hypotheses. 2020;144:109980. 32570163 10.1016/j.mehy.2020.109980 PMC7833987

[40] Mondal R , Deb S , Lahiri D , Shome G . Recurrence of COVID‐19: treading the fine line between relapse and re‐infection. Int J Med Stud. 2020;8(3):311‐313.

[41] To KK‐W , Hung IF‐N , Ip JD , et al. COVID‐19 Re‐Infection by a Phylogenetically Distinct SARS‐Coronavirus‐2 Strain Confirmed by Whole Genome SequencingClinical infectious diseases; 2020. 10.1093/cid/ciaa1275 PMC7499500 32840608

[42] Chen X , Pan Z , Yue S , et al. Disease severity dictates SARS‐CoV‐2‐specific neutralizing antibody responses in COVID‐19. Signal Transduct Target Ther. 2020;5(1):1‐6. 32879307 10.1038/s41392-020-00301-9 PMC7464057

[43] Larson D , Brodniak SL , Voegtly LJ , et al. A case of early re‐infection with SARS‐CoV‐2. Clin Infect Dis: An Official Publ Infect Dis Soc Am. 2020.

[44] Iwasaki A . What reinfections mean for COVID‐19. Lancet Infect Dis. 2021;21(1):3‐5. 33058796 10.1016/S1473-3099(20)30783-0 PMC7550040

[45] Gousseff M , Penot P , Gallay L , et al.Clinical recurrences of COVID‐19 symptoms after recovery: viral relapse, reinfection or inflammatory rebound? J Infect. 2020;81(5):816‐846. 10.1016/j.jinf.2020.06.073 PMC7326402 32619697

[46] Chen Z , Wherry EJ . T cell responses in patients with COVID‐19. Nat Rev Immunol. 2020;20(9):529‐536. 32728222 10.1038/s41577-020-0402-6 PMC7389156

[47] Zhao J , Yuan Q , Wang H , et al. Antibody responses to SARS‐CoV‐2 in patients with novel coronavirus disease 2019. Clin Infect Dis. 2020;71(16):2027‐2034. 32221519 10.1093/cid/ciaa344 PMC7184337

[48] Liu A , Li Y , peng J , Huang Y , Xu D . Antibody responses against SARS‐CoV‐2 in COVID‐19 patients. J Med Virol. 2021;93(1):144‐148. 32603501 10.1002/jmv.26241 PMC7362084

[49] Iyer AS , Jones FK , Nodoushani A , et al. Persistence and decay of human antibody responses to the receptor binding domain of SARS‐CoV‐2 spike protein in COVID‐19 patients. Sci Immunol. 2020;5(52). 10.1126/sciimmunol.abe0367 PMC7857394 33033172

[50] Baumgarth N , Nikolich‐Žugich J , Lee FEH , Bhattacharya D . Antibody responses to SARS‐CoV‐2: let us stick to known knowns. J Immunol. 2020;205(9):2342‐2350. 32887754 10.4049/jimmunol.2000839 PMC7578055

[51] Amanna IJ , Carlson NE , Slifka MK . Duration of humoral immunity to common viral and vaccine antigens. New Eng J Med. 2007;357(19):1903‐1915. 17989383 10.1056/NEJMoa066092

[52] Zhang J , Ding N , Ren L , et al. COVID‐19 Reinfection in the Presence of Neutralizing AntibodiesNational Science Review; 2021. 10.1093/nsr/nwab006 PMC7928639 34676097

